# The therapeutic target of estrogen receptor-alpha36 in estrogen-dependent tumors

**DOI:** 10.1186/1479-5876-12-16

**Published:** 2014-01-21

**Authors:** Yu Gu, Tianxiang Chen, Elena López, Weizhu Wu, Xiangdong Wang, Jiang Cao, Lisong Teng

**Affiliations:** 1 Department of Surgical Oncology, The 1st Affiliated Hospital, School of Medicine, Zhejiang University, 79, Qingchun Road, Hangzhou 310003 Zhejiang Province, China; 2 Department of Thoracic Surgery, The 1st Affiliated Hospital, School of Medicine, Zhejiang University, Hangzhou, Zhejiang Province, China; 3 Hospital Universitario Niño Jesús, Av. Menéndez Pelayo 65, Madrid 28009, Spain; 4 Department of Breast Surgery, Lihuili Hospital, School of Medicine, Ningbo University, Ningbo, Zhejiang Province, China; 5 Department of Respiratory Medicine, The First Hospital of Wenzhou Medical University, Wenzhou, China; 6 Clinical Research Center, The 2nd Affiliated Hospital, School of Medicine, Zhejiang University, Hangzhou, Zhejiang Province, China

**Keywords:** Estrogen receptor, ER-alpha36, Non-genomic signaling, Breast cancer, Hormone-dependent cancer, Endocrine therapy resistance

## Abstract

Estrogen receptor-alpha36 (ER-α36) is a new isoform of estrogen receptors without transcriptional activation domains of the classical ER-α(ER − α66). ER-α36 is mainly located in cytoplasm and plasma membrane. ER-α36 mediates non-genomic signaling and is involved in genomic signaling of other ERs. Recently ER-α36 is found to play a critical role in the development of estrogen-dependent cancers and endocrine resistance of breast cancer. The present article overviews and updates the biological nature and function of ER-α36, potential interaction of ER-α36 with other estrogen receptors and growth factor receptors, intracellular signaling pathways, potential mechanism by which ER-α36 may play an important role in the development of tumor resistance to endocrine therapies.

## Introduction

Estrogens, mainly 17β-estradiol (E2), regulate growth, differentiation, and homeostasis of eukaryotic cells. Notably, it is associated with a higher risk of the development of breast and endometrial cancer [1]. Two forms of estrogen receptors (ERs), ER-α and ER-β, were respectively discovered in 1962 [2] and in 1996 [3], and suggested to dominate the regulation of various biological functions. A number of ER variants were identified since then and found to coexist with these wild-type ERs, including ER-α36, ER-α46, and ER-β2-5 [4-6]. Of them ER-α36 is a 36-kDa novel isoform of ER-α66 identified and cloned by Wang et al., in 2005 [6]. It is considered as a new and important factor to understand the pleiotropic effects of estrogen, even in organs without ER-α66. Human ER-α36 differs from hER-α66 by lack of both transcriptional activation domains (AF-1 and AF-2), but it retains partial dimerization, DNA-binding and ligand-binding domains. Initially ER-α36 was proposed to be a dominant-negative effector in estrogen-stimulated activation of estrogen-responsive genes through hER-α66 [6] and crucial in estrogen-stimulated membrane responses [7].

The intracellular location is important for molecular function of ERs. With different intracellular expression on nucleus, cytoplasmic or membrane, estrogens can function through ERs differently [8]. The genomic action of ER is considered as nuclear-initiated steroid signaling, and the non-genomic action of ER as membrane-initiated steroid signaling [9-11]. Non-genomic pathway regulates more genes than just genomic action of ER alone. It involves in different cellular processes like proliferation, survival, apoptosis and differentiated functions in diverse cell-types. ER-α36 is found localized in both plasma membrane and cytoplasm. Thus, it may be related with both genomic and non-genomic signaling network.

The activation of ERs was found to be associated with carcinogenesis, progression, and endocrine resistance of steroid-responsive cancers [12]. The expression of ER-α36 was correlated with clinical phenotypes and endocrine therapy responses of patients with various cancers, particularly breast cancer. It was proposed that ER-α36 as a novel tumor-associated ER isoform could act as a potential biomarker for diagnosis and treatment of estrogen-dependent carcinoma [4,6]. Signaling pathways activated by estrogen and anti-estrogen through ER-α36 may help us understand why human breast cancers are resistant to or worsened by anti-estrogen therapy. The present review surveys updated knowledge on ER-α36 biology, non-nuclear receptor functions, its role in estrogen-dependent tumors and its action in human breast cancer diagnosis and treatment, especially emphasize on endocrine resistance.

### The biology of ER-α36

**Figure 1:**
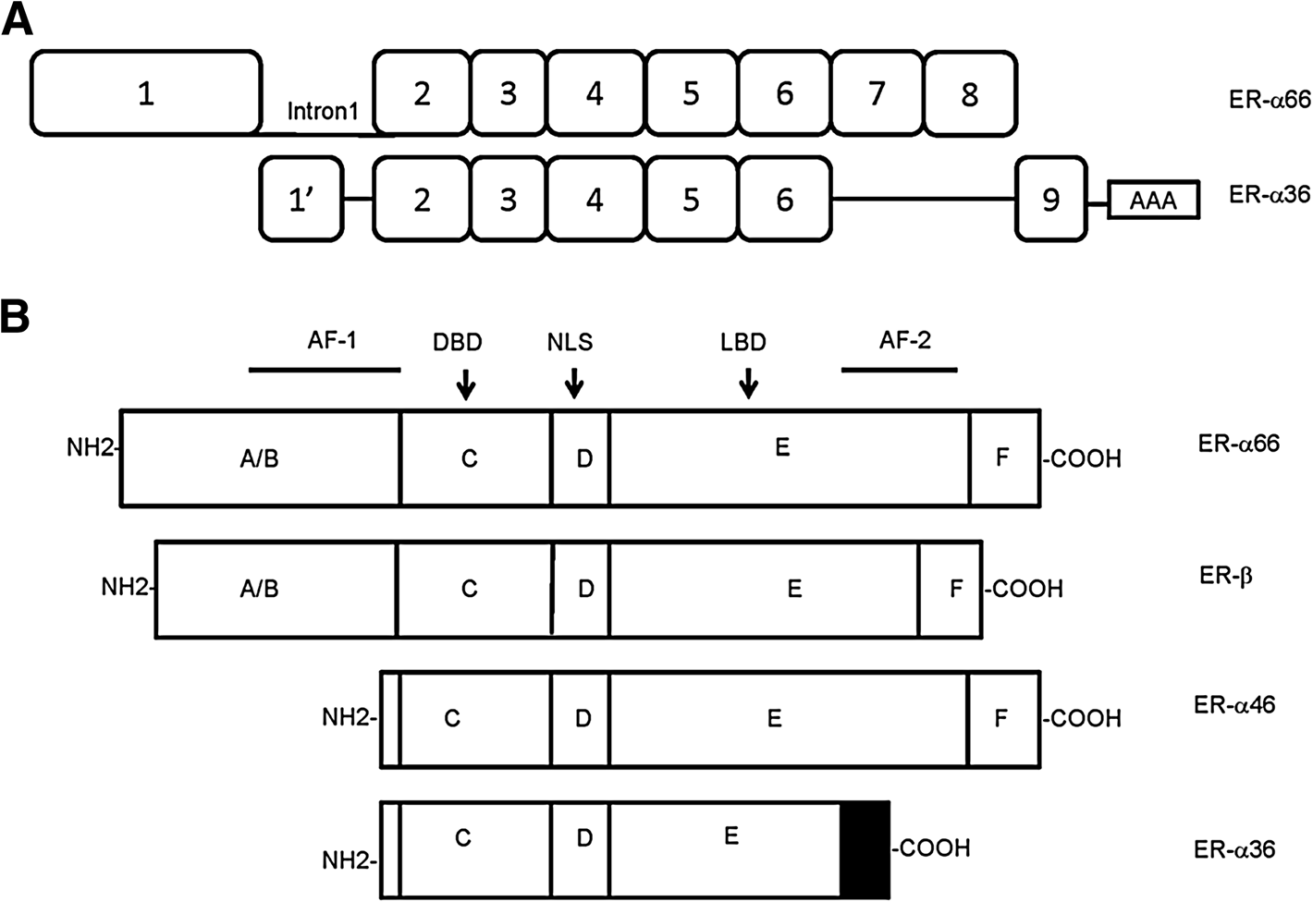
**Biological structure of ER-a36. (A)** The exon of ER-α36 from intron1 of ER-α66 gene is designated as 1′. The extra exon of ER-α36 gene that is beyond 8 exons of ER-α66 gene is numbered as 9. ER-α36 has a unique 27 amino acid domain at C-terminus. Deletions are indicated by a single line. **(B)** Protein structure of ER isoforms includes A/B domain contains transcriptional-activation function AF-1, C domain represents DNA-binding-domain (DBD), D domain contains nuclear localization signal (NLS), and E domain corresponds to ligand binding domain (LBD) and transcriptional-activation function AF-2. ER-α36 lacks AF-1 and AF-2. The last 27 amino acids of ER-α36 are indicated by a shaded box.

The non-coding novel exon of ER-α36 from the first intron of ER-α66 gene is designated as ‘exon1’, to distinguish it from the original exon1 in ER-α66 gene. ‘Exon1’ is directly spliced into exon2 of ER-α66 gene. ER-α36 continues from exon2 to exon6 of ER-α66 gene with 100% match. It has a unique C-terminal 27 amino acid domain that replaces the last 138 amino acids encoded by exon7 and 8 of the ER-α66 gene [6] (Figure 1A). ER-α66 and ER-β are composed of three independent but interacting functional domains [13,14], among which the A/B domain contains AF-1 and is involved in interactions with co-activators and transcriptional activation of target genes [15]. The DNA binding domain, or C domain allows both receptors to bind to similar target sites. The D domain or hinge region contains nuclear localization signal. The E/F region is a ligand-binding domain that mediates ligand binding, receptor dimerization, nuclear localization and ligand-dependent transactivation (AF-2) [8]. Compared to ER-α66, ER-α36 lacks two transcriptional activation domains AF-1 and AF-2 while retains the DNA-binding domain as well as partial dimerization, ligand-binding domains (Figure 1B) [6]. ER-α36 elicits membrane-initiated signaling in response to E2-α, E2-β, E3, and E4 as well as tamoxifen. It indicates that ER-α36 possesses a broader ligand-binding spectrum than ER-α66 and it may act as a potential mediator of mitogenic estrogen signaling [7].

**Figure 2:**
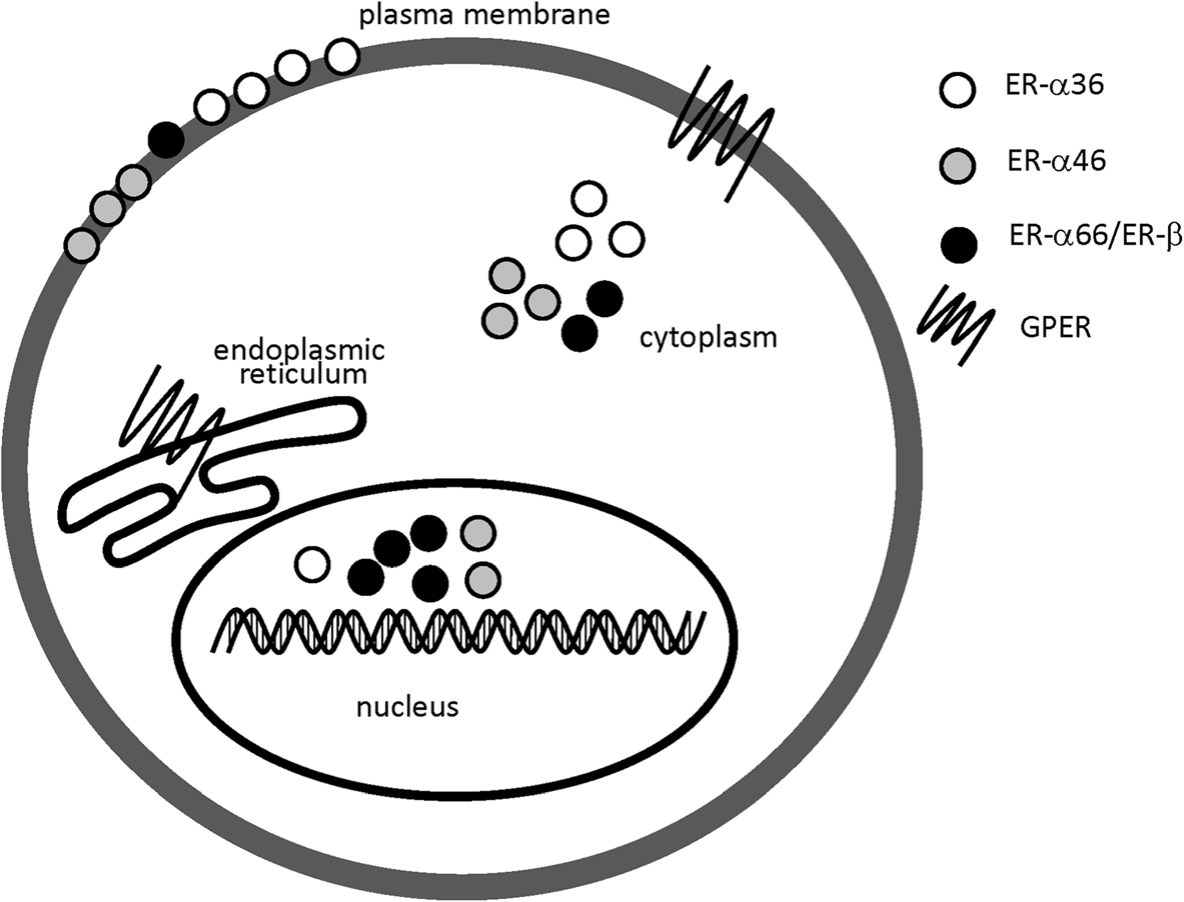
**Diagram of locations of different ERs.** ER-α36 is mainly localized in plasma membrane and cytoplasm, little is found in nucleus. Classical ERs like ER-α66 and ER-β are mainly expressed in nucleus and cytoplasm with little in plasma membrane. As to other ERs (e.g. ER-α46 and GPER), the cellular localization usually depends on cell types.

Wang et al. isolated nuclear, plasma membrane and cytosolic fractions from ER-α36-expressing HEK-293 cells and found that 50% of ER-α36 fractionates with plasma membrane, 40% with cytosol and 10% with nuclei [7]. The variation of ER-α36 locations on plasma membrane or cytoplasm was noted to be associated with different types of cancer cells including breast [16], endometrial [17], colorectal [18], gastric [19] and hepatic cancers [20]. ER-α36 is also expressed in the cell membrane of normal hamster ovary cells [21]. Furthermore, ER-α36 is modified by post-translational palmitoylation in the ligand-binding domain and it has three potential myristoylation sites that may contribute to its membrane localization [6,22] (Figure 2).

### The non-nuclear receptor functions of ER-α36

**Figure 3:**
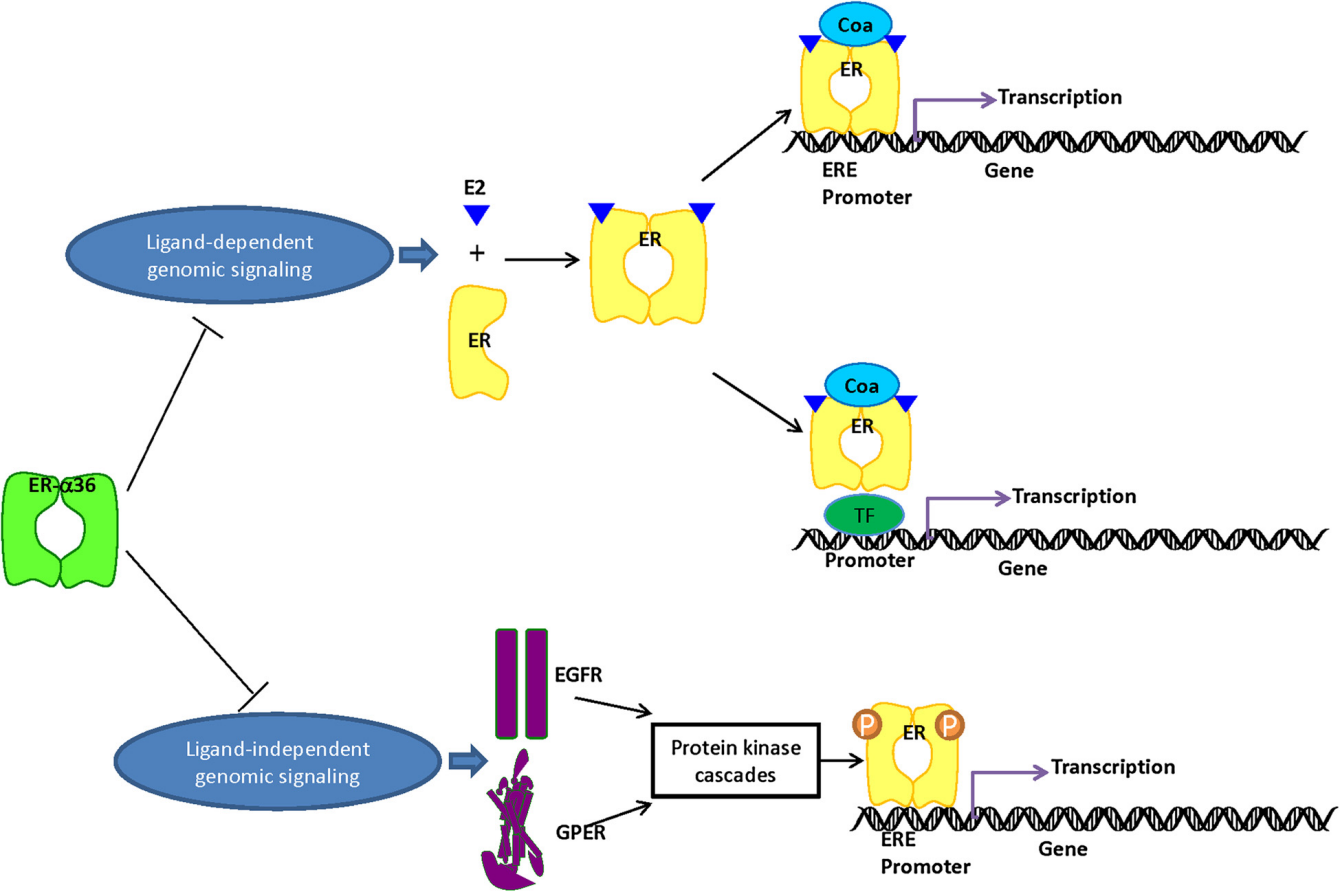
**The genomic activity signaling of nuclear estrogen receptors (ERs) can be inhibited by ER-α36.** The classical pathway of estrogen signaling is ligand-activated ERs bind specifically to estrogen response elements (EREs) in the promoter of target genes. The ligand-dependent indirect genomic regulation of gene transcription includes interactions with other transcription factors (TF). In ligand-independent pathway, ERs can be stimulated by other signaling pathways, such as growth factor signaling which eliciting genomic effects in the absence of ligands. In this case, activated kinases phosphorylate ERs, it thereby activate them to dimerize, bind DNA and regulate genes. ER-α36 can suppress both estrogen-dependent and estrogen-independent transactivation functions which signaling through nuclear ERs like ER-α66 and ER-β.

The classical mechanism of ER action involves the binding of estrogen to nuclear receptors. After that the receptors dissociate from Hsps, dimerize and bind to specific response elements known as estrogen response elements which located in the promoters of target genes [23]. ERs can mediate the transcription through protein-protein interactions with other DNA-binding transcription factors in the nucleus. In addition, ligand-independent pathways have been described. Growth factor signaling can trigger ERs through the activation of kinases and/or be associated co-regulators in the absence of ligand [24] (Figure 3).

E2 was reported to bind to a cell surface receptor and stimulate a rapid generation of cAMP [25], meanwhile the plasma membrane-localized ER was also proposed to transduce membrane-initiated estrogen signaling. Estrogens can rapidly function through the non-genomic mechanisms independent on the activation of RNA and protein synthesis. As a non-nuclear ER, ER-α36 not only inhibits the genomic estrogen signaling of nuclear ER, but also mediates the non-genomic estrogen signaling.

### ER-α36 and other estrogen receptors

ER-α36 lacks transcription activation domains of ER-α66 and detectable levels of intrinsic transcriptional activity with or without the presence of E2 [7]. The presence of ER-α36 could inhibit E2β-dependent and -independent transactivation functions mediated by AF-1 or AF-2 domains of ER-α66 and ER-β (Figure 3). ER-α36 also effectively competes with ER-α66 and ER-β for the DNA-binding elements in estrogen-responsive genes [7]. On the other hand, transient co-transfection experiments demonstrated that ER-α66 suppressed ER-α36 promoter activity in an estrogen-independent manner, the suppression could be released by ER-α36 itself [26]. Wilm’s tumor1, as a dual transcription factor, was found to regulate the promoter activity of ER-α66 and ER-α36 oppositely in breast cancer cells [27]. All above may explain the phenomenon that ER-α36 expression appears to be associated with decreasing ER-α66 expression in many kinds of cancers [16].

**Figure 4:**
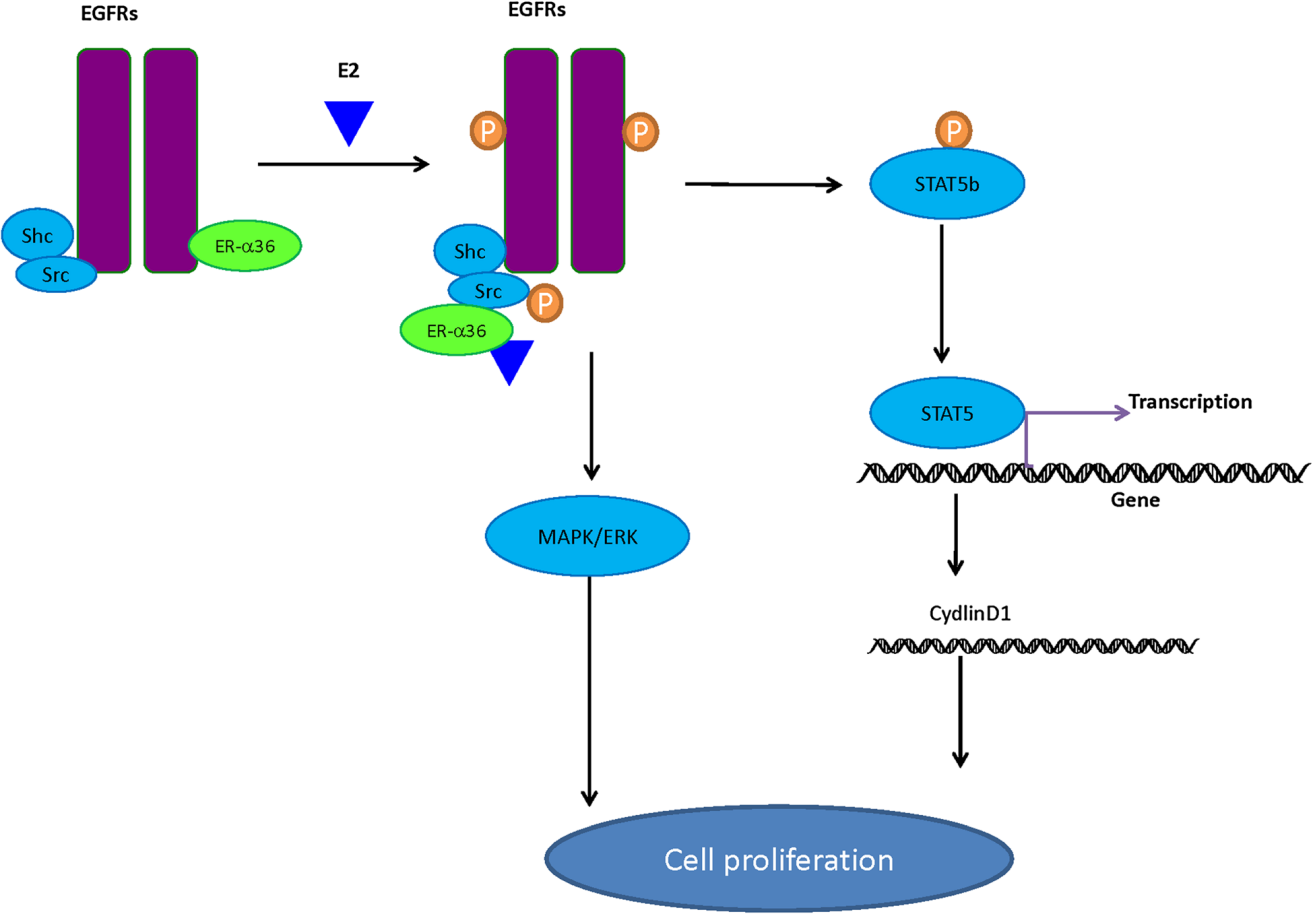
**Interactions between ER-α36 and EGFR in mitogenic signaling.** After treatment with estrogen, ER-α36 is dissociated from epidermal growth factor receptor (EGFR) and is associated with Src and Shc. Furthermore, E2 induces auto-phosphorylation of Src and Src-dependent phosphorylation of EGFR. EGFR activates signal transducer and activator of transcription 5(STAT5), which leads to transcription of target genes such as Cyclin D1*.* On the other hand, ER-α36 and EGFR complex mediated non-genomic signaling like MAPK/ERK pathway is also involved in the signaling pathway contributing to cell proliferation.

A new membrane-bound estrogen receptor GPER was identified with homology to the G-protein-coupled receptor superfamily. In addition, GPER was suggested to be associated with classical estrogen receptor expression in breast cancer [28-30]. Kang et al. found that G1, a GPER-specific agonist, could stimulate ER-α36 to regulate the non-genomic signaling pathway through p-ERK1/2 rather than GPER [31]. GPER was also found to be necessary for the stimulated expression of ER-α36 which triggered by E2 [32]. Current knowledge suggests estrogen may activate GPER to induce ER-α36 expression (as shown in Figure 4).

### ER-α36 and growth factor receptors

Both ER-α36 and EGFR are mainly localized on plasma membrane, they have a positive correlation with each other in breast cancer and endometrial cancer [33]. EGFR signaling might induce the promoter activity of ER-α36 gene via an Ap-1 binding site. ER-α36 was found to be necessary for both EGFR membrane localization and E2-mediated stimulation of EGFR expression in TCam-2 cells. It stabilized the steady state protein levels of EGFR in breast cancer [32,34]. After E2-β treatment of breast cancer cells, ER-α36 gradually dissociates from EGFR and meanwhile associates with Src and Shc. This process suggests that ER-α36 might dynamically change its partners within EGFR/Src/Shc complex during estrogen signaling [34]. Interestingly, low concentration of estrogen or certain anti-estrogens like tamoxifen are shown to stimulate cell proliferation by eliciting mitogenic signaling pathway, while high concentration to inhibit cell growth [35,36]. Such paradoxical effect was believed to be regulated by ER-α36 in ER-negative breast cancer cells [34,35]. Furthermore, ER-α36 is also proposed to regulate the phosphorylation of both Src/EGFR and MAPK/ERK during mitogenic signaling and to activate Cyclin D1 promoter activity through Src/EGFR/STAT5 pathway (Figure 4). EGF treatment was discovered to increase ERK1/2 phosphorylation in ER-α36-expressing Hec1A cells, but not in ER-α36 knockdown cells. This finding tells us ER-α36-EGFR complex mediated MAPK/ERK pathway activation may be critical in the non-genomic estrogen signaling [17]. Recently we found, for the first time, that ER-α36 up-regulated EGFR expression, while down-regulated ER-α66 expression in MCF7 cells. Our study provided a potential mechanism for the growth switch of breast tumors after acquired tamoxifen resistance [37].

Human epidermal growth factor receptor 2 (HER2), as member of EGFR superfamily, was also significantly correlated with ER-α36 expression in patients with breast cancer like EGFR [38]. In vitro study showed HER2 and ER-α36 was present in the same protein complex in ER-negative breast cancer SK-BR-3 cells. It was noted that HER2 signaling activated ER-α36 promoter activity through an AP-1-dependent signaling pathway and ER-α36 activated HER2 transcription [39]. Therefore, The interplay between growth factor receptors and ER-α36 may play an important role in development and progression of subsets of cancer with highly expression of ER-α36.

### ER-α36 and downstream kinases

#### Mitogen-activated protein kinases

The signaling cascades in the MAPK/ERK pathways are proposed as major intracellular communication in breast, prostate and colon cancers [40]. Wang et al. reported that ERK1/2 phosphorylation of ER-α36 transfected HEK293cells was increased comparing to control cells with the E2β treatments or not. Similar finding was introduced after cells treating with E2β-BSA which was a membrane-impermeable form of E2-β [7]. It indicates that E2-mediated ERK1/2 activation might be initiated by a membrane–initiated estrogen-signaling pathway via ER-α36. Importantly, such mechanism was also proven in breast and endometrial cancer cells [31,34,36,41,42]. Those findings demonstrate the involvement of MAPK/ERK pathway in estrogen-related signal of hormonal dependent cancer cells by a combination of ER-α36. Besides, PKC was evidenced to rapidly enhance phosphorylation of proliferation promoting proteins by activation of ERK1/2 [43]. Tong et al. found that ER-α36 mediated E2-induced activation of MAPK/ERK pathway also via PKC in endometrial cancer cells [44]. Furthermore, the stimulated PKC of ER-α36 expressing breast cancer cells contributed to increased proliferation in response to E2 [45].

**Figure 5:**
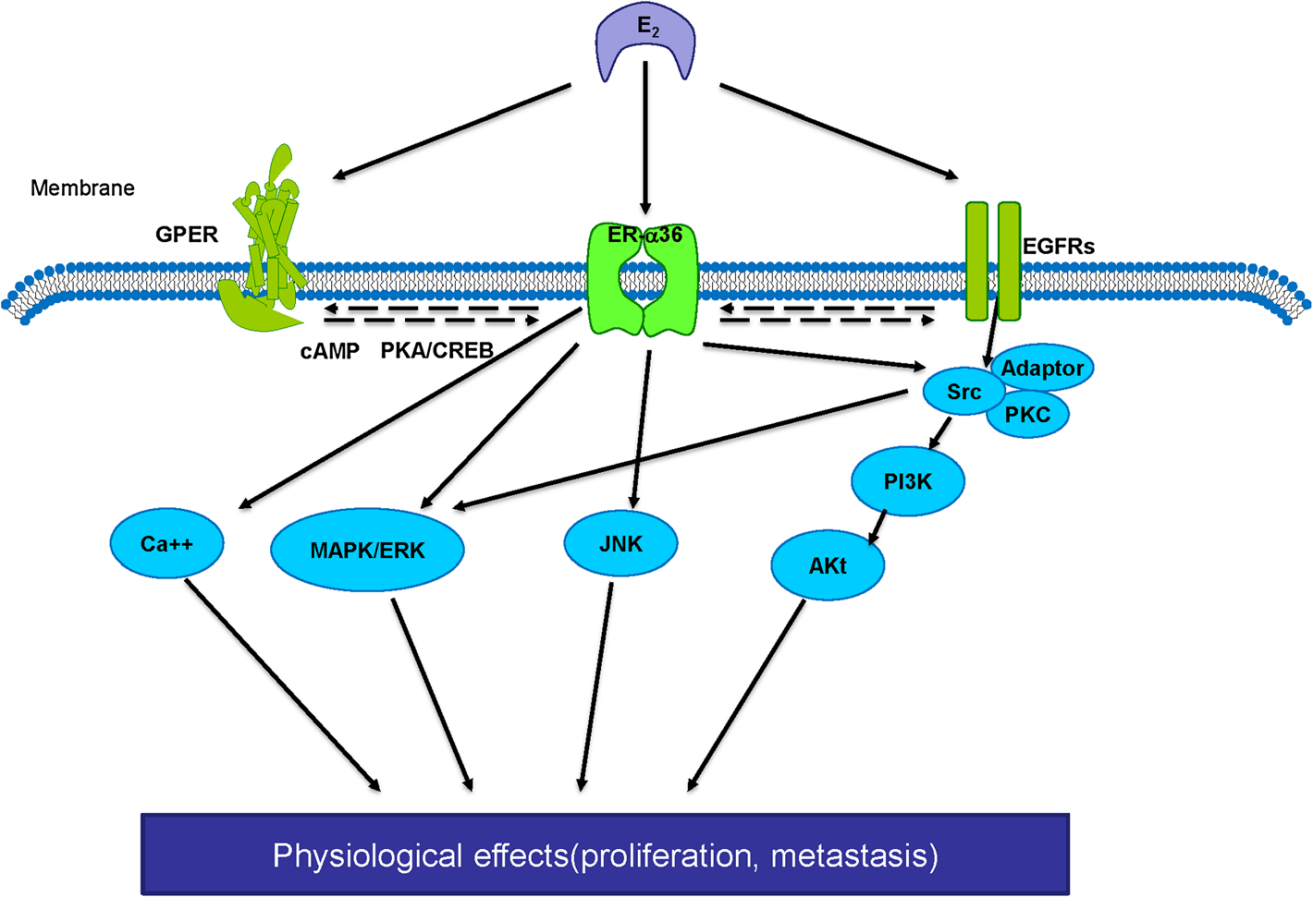
**The non-genomic effects of ER-α36.** ER-α36 activated by E2 interacts with adaptor proteins (adaptor) and downstream signaling molecules such as c-Src, which mediates rapid signaling via PI3K/Akt and MAPK/ERK pathways. ER-α36 also turns on other downstream kinases like c-Jun N-terminal kinases (JNK) and intracellular Ca2+ mobilization. The interactions between ER-α36 and EGFR/GPER are also involved in the non-genomic signaling.

Only with the presence of ER-α36 but not ER-α66, the treatment of estradiol and anti-estrogenic agents led to rapid activation of p-ERK1/2 and substantial increase of cell migration and invasiveness in inflammatory breast cancer [42]. Both basal and ligand-induced migration and invasiveness of ER-α36 expressing breast cancer cells were drastically reduced after treatment of MEK inhibitor U0126.These results implicated that phosphorylation of ERK1/2 by MEK might be involved in the cell motility and invasiveness. It was also evidenced by the up-regulation of p-ERK1/2 in patients with inflammatory breast cancer [42]. Collectively it is possible that ER-α36 may promote proliferation and invasion of cancer cells via MAPK/ERK signaling pathway.

c-Jun N-terminal Kinases (JNKs), as another principal members of MAPK family, regulate cell proliferation, differentiation and migration [46]. Our laboratory revealed that less activation of JNKs and major proportion of cells arrested at the G2/M phase in the absence of ER-α36 were seen after treatment of paclitaxel [47], which induces cell cycle arrest at the G2/M phase and result in endoreduplication [48]. Our results suggest that ER-α36 antagonizes the effect of paclitaxel via activation of JNKs pathway (Figure 5).

#### The serine/threonine kinase

The serine/threonine kinase (Akt), also named as protein kinase B, is important in cell proliferation and survival by inhibition of apoptosis [49]. Treatments with testosterone, E2, or tamoxifen induce rapid phosphorylation of Akt in Hec1A cells can be abrogated in the absence of ER-α36 [41]. Tamoxifen induces Akt phosphorylation in ER-α36 high expressing MCF-7cells, while pre-treatment with PI3K inhibitor abrogates Akt phosphorylation stimulated by E2 or tamoxifen. It indicates that ER-α36 may mediate multi-ligands-induced Akt phosphorylation mainly through PI3K pathway [36]. The PI3K/Akt signaling pathway in an ER-α36-dependent way could be activated by the inhibition of Caveolin-1, a major protein component of Caveolae and a potential tumor suppressor [50-52] (Figure 5). In addition, E2 fail to induce intracellular Ca2^+^ mobilization in ER-α36-negative breast cancer cells. It suggests that ER-α36 is involved in estrogen-induced intracellular Ca2^+^ response, although the exact mechanisms by which ER-α36 influences Ca2^+^ mobilization remain unclear [31].

### ER-α36 and estrogen-dependent tumors

#### Breast cancer

##### General characteristics of ER-α36 in breast cancer

ER-α36 is both expressed on plasma membrane and in cytoplasm in different breast cancer cell lines, the expression of which is associated with a decrease of ER-α66 in nuclear and/or cytoplasm [7,16,26]. However, ER-α36 is not presented in normal mammary epithelial cells like MCF10A [26,39]. The depletion of ER-α36 via siRNA can induce apoptosis of ER negative breast cancer cells [53]. The migration and invasion activity of breast cancer cells can be inhibited in the absence of ER-α36 [47]. ER-α36 rapidly activates PKC in response to E2, which leads to promotion of proliferation, protection against apoptosis, and metastasis in breast cancer cells with or without ER-α expression [45]. It suggests that ER-α36 may be a potential therapeutic target for breast tumor growth and metastasis. Different from mRNA expression, only a few translated protein products of ER-α66 variants have been found naturally in breast cancer like ER-α36 [54-57]. ER-α36 was reported to express in a cytoplasmic and plasma-membrane-associated pattern in human tissue of both ER-α66-positive and -negative breast cancer. High expression of ER-α36 is more common in ER-α66-negative tumors, whereas low expression of ER-α36 is more frequently seen in ER-α66-positive tumors [16,33,34,47,58]. In addition, ER-α36 was also found within Golgi apparatus reflecting a putative implication in protein synthesis.

The presence of ER-α36 is not limited to tumor cells of breast cancer patients, but also in endothelial cells, adipocytes, infiltrating lymphocytes and fibroblasts in tumor microenvironment. Moreover, the expression of ER-α36 could be elevated in some cases with strong desmoplasia at the time fibroblasts become reactive [58]. Membrane ER-α36 is correlated with the expression of pro-angiogenic miRNA210 in an inverse manner and stratifies miR210-related patients survival, this may be understood as a possible anti-angiogenic effect of ER-α36 [58]. Lower expression of ER-α36 is positively correlated with larger size of the tumor, metastases to lymph nodes, advanced severity of disease, and shorter survival of patients with breast cancer [58,59], of which detail information is summarized in Table 1.

**Table 1:** Expression of ER-α36 in cancers

Tumor type (*)	**Study**	**Kind/num. of analyzed samples**	Method (**)	**Potential clinical significant**
BC	Lee, *et al.*[16]	37 BC tissues	IHC/WB	Inverse association with ER-α66 expression
BC	Shi, *et al.*[38]	896 BC tissues	IHC	ER-α66+/ER-α36Hi tumors are less likely to benefit from tamoxifen treatment than ER-α66+/ER-α36- tumors
BC	Zheng, *et al.*[59]	74 pairs of BC tissues and matched normal tissues	PCR	Lower expression in BC tissues than in matched normal tissues.
				Inverse association with local progression/lymph node metastasis/advanced cancer stage.
BC	Zhang, *et al.*[34]	12 triple-negative BC tissues	IHC	Expressed in triple-negative BC
BC	Pelekanou, *et al.*[58]	49 triple-negative BC tissues % 34 matched normal samples	IHC	Highly expressed in triple negative BC.
				Positive association with overall survival rate
BC	Vranic, *et al.*[33]	19 pure apocrine carcinomas and 11 adenoid cystic carcinomas of the breast	IHC	Highly expressed in ER-α66 negative apocrine and adenoid cystic carcinomas of the breast.
				Higher expression in the malignant epithelium than adjacent normal breast tissue
BC	Zhang, *et al.*[35]	20 BC tissues	IHC	Inverse association with ER-α66 expression
EC	Tu, *et al.*[17]	45 EC tissues	IHC	Positive association with high-stage/high-grade
EC	Sun, et al. [60]	73 EC tissues, 20 normal endometrial tissues % 9 atypical endometrial hyperplasia	IHC	Lower expression in EC tissues than in normal endometrial tissues and atypical hyperplasia.
				Inverse association with disease-free survival rate
HCC	Miceli, *et al.*[20]	5 normal liver tissues,8 liver cirrhosis specimens % 8 HCC tissues	PCR	Inverse association with ER-α66 expression.
				Increase in a stepwise manner from non-tumoural tissues, cirrhotic tissues to HCC tissues.
CRC	Jiang, *et al.*[18]	35 CRC tissues and their matched normal tissues	PCR	Lower expression in CRC than matched normal tissues.
				Inverse association with tumour stage/lymph node metastasis.
GC	Deng, *et al.*[61]	22 GC tissues	IHC	Higher expression in GC than paired normal tissues.
				Positive association with lymph node metastasis
GC	Wang, *et al.*[19]	45 GC tissues and matched normal tissues	PCR	Lower expression in GC than normal tissues

(*) Tumor type: BC: Breast Cancer, EC: Endometrial Cancer, HCC: Hepatocellular Carcinoma, CRC: Colorectal Cancer, GC: Gastric Cancer.

(**) Method: WB: Western blotting, IHC: Immunohistochemistry, PCR: Polymerase chain reaction.

##### Therapeutic significance

Anti-estrogens (e.g. tamoxifen, ICI182,780) and aromatase inhibitors (e.g. anastrazole, letrozole) are widely used for the treatment of breast cancer, especially ER-positive breast cancer. Clinical studies demonstrated that approximately 40% of patients with ER-α66-positive breast cancer had higher expression of ER-α36, and were less sensitive to TAM treatment in comparison with those with ER-α66+/ER-α36-tumors [38]. ER-negative breast cancer is less or even non-responsive to anti-estrogen therapy. However, 45% patients with ER-/PR + breast cancer and 10% with ER-/PR-cancer responded to tamoxifen treatment [62]. Gu et al. found that ER-α66-deficient mice retained rapid estrogen-stimulated membrane effects in neurons which were not blocked by ICI 182,780. ER-α66−/−mice were created by an insertional disruption of the first coding exon of mouse ER-α66 gene. This exon is the one skipped in the generation of transcripts of ER-α36 [63]. Thus, it is possible that ER-α36 may play an important role in the de novo and acquired resistance of breast cancer patients to endocrine therapy.

TAM and its metabolites 4-hydroxytamoxifen (4-OHT) act as antagonists of estrogen by competing with estrogens for the ligand binding domain of ER [64]. It was reported that TAM and 4-OHT failed to block but rather to stimulate the estrogen-triggered ERK1/2 activation through ER-α36 [7]. Later it was found that tamoxifen promoted proliferation of endometrial cancer cells through ER-α36-mediated activation of MAPK/ERK and PI3K/Akt pathways and ER-α36 overexpression led to tamoxifen resistance in MCF-7 cells [36]. Let-7 microRNAs can induce sensitivity of breast cancer to tamoxifen by down-regulation of ER-α36 signaling [65]. It is suggested that ER-α36 and its non-genomic activities may be involved in de novo resistance to tamoxifen and even promote the agonist action of tamoxifen [7].

**Figure 6:**
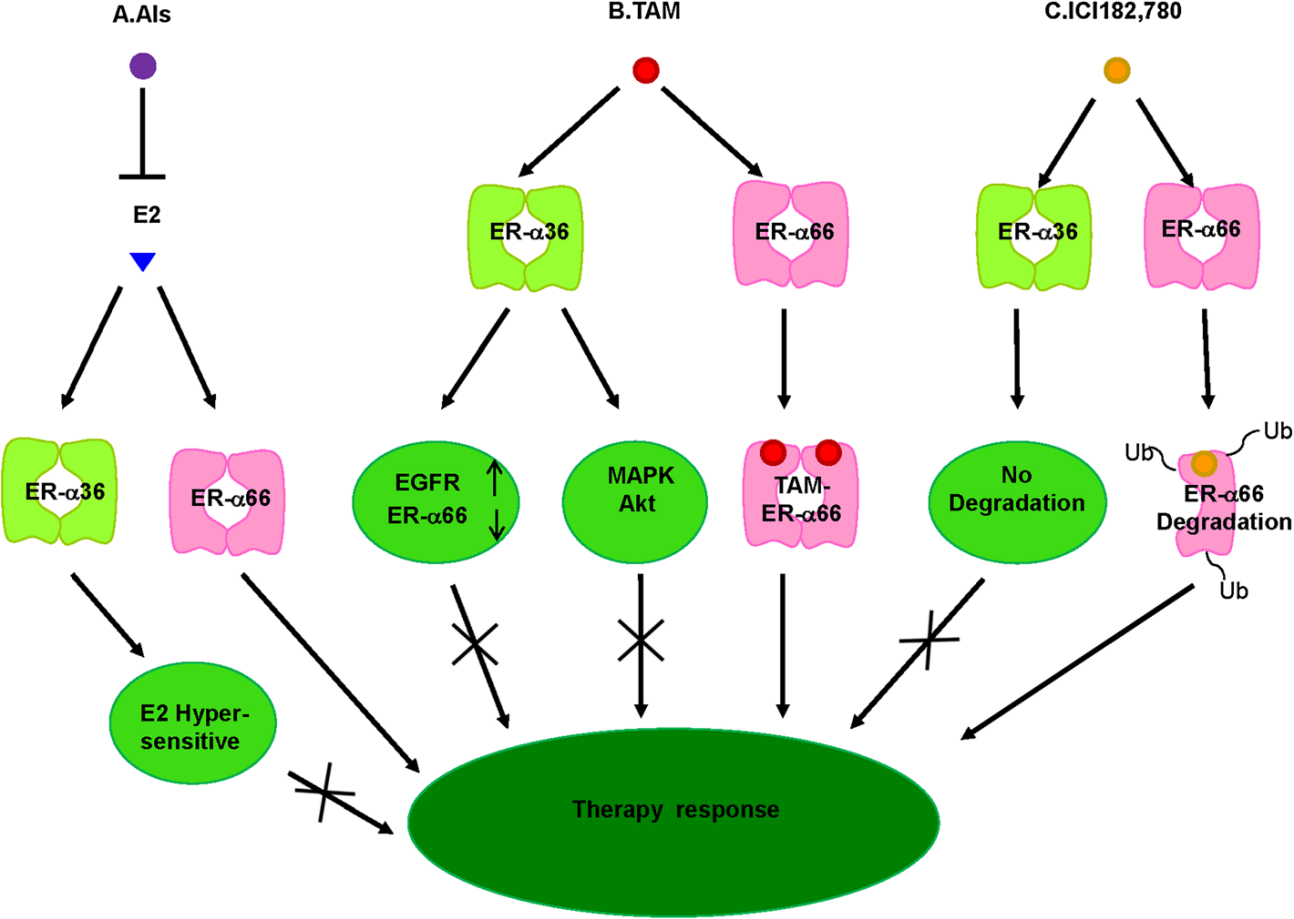
**ER-α36 and endocrine therapy resistance. (A)**. Aromatase inhibitors (AIs) inhibit the synthesis of estrogen. High expression of ER-α36 contributes to estrogen hypersensitivity. This provides an explanation for failure of AIs treatment in ER-negative breast cancer patients. **(B)**. Tamoxifen (TAM) inhibits ER-mediated mitogenic estrogen signaling by competing with estrogens (E2) for the ligand binding domain of ER-α66. The non-genomic activities of ER-α36 are involved in de novo TAM resistance of breast cancer. Up-regulation of EGFR expression and down-regulation of ER-α66 expression by ER-α36 could contribute to generation of acquired TAM resistance. **(C)**. ICI 182,780 can accelerate degradation of ER-α66 protein so as to inhibit mediated estrogen signaling. However, ICI182,780 fails to induce degradation of ER-α36 to take therapeutic effect. AIs: Aromatase inhibitors TAM: Tamoxifen

Advanced studies of ER biology have highlighted the intimate cross talk between ER and HER2/growth factor signaling pathways to be a fundamental contributor to the development of TAM therapies resistance. Overexpress of HER2 is found in 25-30% of breast cancers [66] and it is related to less responsive to tamoxifen treatment [67]. ER-α36 and HER2 are demonstrated to positively regulate the interaction and expression of each other in breast cancer [38,39]. It is possible that certain signaling pathway mediated by HER2 activates ER-α36 expression, which then confers tamoxifen resistance of HER2 over-expressing tumors. On the other hand, our earlier study contributed further knowledge of interactions between ER and EGFR during development of TAM resistance. We established a TAM-resistant breast cancer cell line MCF-7/TAM, of which ER-α36 and EGFR were both overexpressed while ER-α66 was down-regulated comparing to parental MCF-7 cells. The silence of ER-α36 expression of MCF-7/TAM cells resulted in decreased expression of EGFR, increased expression of ER-α66, reduced proliferation rate together with decreased in vitro migratory and invasive ability. ER-α36-transfected MCF-7 cells could increase EGFR expression and decrease ER-α66 expression which lead to a decreased sensitivity to TAM. These results indicate, for the first time, a regulatory role of ER-α36 in up-regulation of EGFR expression and down-regulation of ER-α66 expression. This may be a potential mechanism by which the cells acquired TAM resistance [37] (Figure 6B).

Fulvestrant (ICI 182,780), a potent anti-estrogen, inhibits estrogen signaling after it binds to ER-α66 and mediates downstream molecular activities. Through these biological processes, ICI 182,780 impairs ER-α66 dimerization, inhibits nuclear localization of receptor, and accelerates the degradation of ER-α66 protein without any reduction of ER-α66 mRNA [68-70]. However, it was reported that ICI182,780 failed to induce degradation of ER-α36 [71], probably due to truncated ligand-binding domain of ER-α36 which lacked the helices 9-12 of ER-α66 [6].The helix-12 domain is critical in protein degradation induced by ICI182,780, different positioning of helix12 and F domain of ER-α66 could regulate various functions between agonists and antagonists [72,73]. It may provide a molecular explanation for the failure of ICI182, 780 to block the non-genomic estrogen signaling mediated by ER-α36 in ER-negative breast cancer [7] (Figure 6C).

The aromatase inhibitors (AIs) like anastrazole and letrozole may deprive estrogen ligands of ER. Aromatases are involved in non-genomic signaling pathway like MAPK/ERK and PI3K/AKt in ER-α36 expressing cells [41]. It was found that anastrozole and letrozole suppressed the plasma level of E2 in breast cancer patients [74]. Breast cancer cells with high expression of ER-α36 can respond to a very low concentration of E2 through the activation of MAPK/ERK signaling pathway [31]. Meanwhile high expression of ER-α36 is proved to induce estrogen hypersensitivity [41]. Therefore, this could be an explanation for the failure of AIs treatment in ER-negative breast cancer (Figure 6A).

ER-negative tumors are often treated with nonspecific cytotoxic chemotherapeutic agents. Studies revealed that taxane-containing chemotherapy yielded a higher overall pathologic complete response rate in patients with ER-negative tumors than in patients with ER-positive tumors [75]. It was indicated that overexpression of ER-α66 decreased the sensitivity of Bcap37 cells (ER-α66 negative) to paclitaxel, suggesting a possible role of ER in chemosensitivity of breast cancer [76]. Our laboratory investigated the possible influence of ER-α36 on the therapeutic effects of paclitaxel in ER-negative breast cancer cells. We found that ER-α36 depletion by microRNA sensitized MDA-MB-231 cells to paclitaxel and the JNK pathway appeared to be involved in the mechanism. It may be a new alternative option to modify or improve therapeutic sensitivity and resistance of breast cancer by blocking ER-α36-mediated non-genomic effects and ER-α36-associated kinase activities.

**Figure 7:**
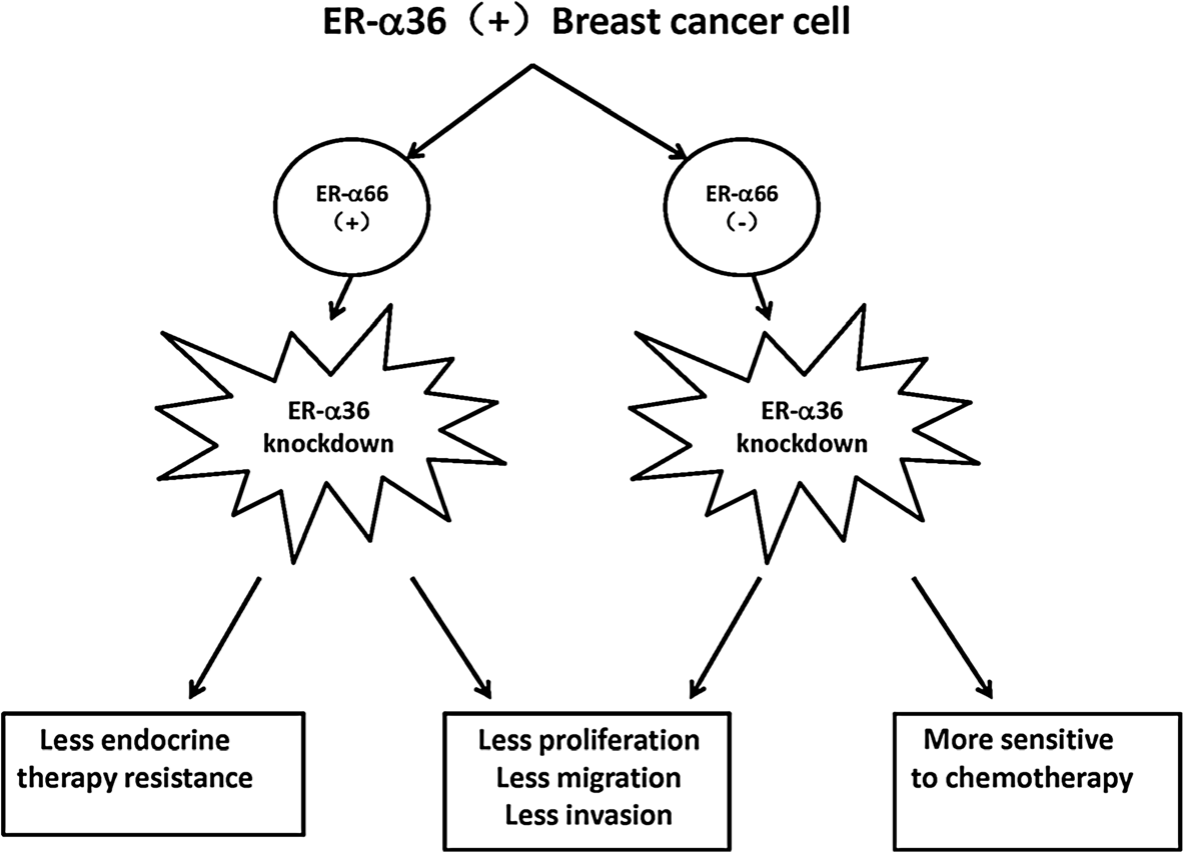
The therapeutic significance of ER-α36 in breast cancer.

According to the findings of our lab and others, ER-α36 knockdown resulted in reduced proliferation rate together with decreased in vitro migratory and invasive ability of breast cancer cells regardless of conventional ER (ER-α66) status. Furthermore, ER-α36 inhibition led to increased chemotherapy sensitivity of ER-negative breast cancer and increased sensitivity to endocrine therapy of ER-positive breast cancer. Thus, targeting strategies against ER-α36 may be a potential treatment for different subtypes of breast cancer (Figure 7).

#### Endometrial cancer

ER-α36 is localized in both plasma membrane and cytoplasm of endometrial cancer cell [17,36,41]. ER-α36-mediated activation of MAPK/ERK and PI3K/Akt pathways promotes proliferation of endometrial cancer cells by different ligands including E2, tamoxifen and testosterone [36,41,44]. In addition, ER-α36 expression is positively associated with advanced clinical stage, pathological grade and poor disease-free survival (DFS) rate of endometrial cancer cases [17]. The expression of ER-α36 in endometrial cancer tissues is significantly lower than that in normal and atypical hyperplasia of endometrial tissues [77]. Thus, ER-α36 may be an important biomarker for diagnosis, prognostication and treatment of endometrial carcinoma (Table 1). It would be even more important if advanced technologies, omics science, systems biology, or clinical bioinformatics, can be applied to investigate dynamic networks and interactions of the ER-α36-specific and dominated elements and understand the molecular mechanisms [78-82].

## Conclusions

As a novel isoform of ER-α66, membrane-associated ER-α36 mainly mediates non-genomic effects of ER regulating various physiopathological processes especially in endocrine resistance of estrogen-dependent tumors. ER-α36 inhibits genomic signaling of nuclear ER and mediates the non-genomic estrogen signaling. Increased expression of ER-α36 is associated with decreased expression of ER-α66 in some cancers. Furthermore, ER-α36 is correlated with larger tumor size, more lymphangiogenesis, more distant metastasis, advanced severity of disease, and poor survival of patients with breast cancer. In all, ER-α36 may act as a critical therapeutic target for diagnosis, prognostication, and personalized treatment of estrogen-dependent tumors.

## Abbreviations

ER: Estrogen receptor; E2: Estradiol; MAPK: Mitogen-activated protein kinase; ERK: Extracellular signal-regulated kinases; JNK: c-Jun N-terminal Kinase; PI3K: Phosphatidylinositide 3-kinase; EGFR: Epidermal growth factor receptor; HER2: Human epidermal growth factor receptor 2; STAT: Signal transducer and activator of transcription; SNCG: Synucleinγ; Cav-1: Caveolin-1; Hsp90: Heat shock protein 90; TAM: Tamoxifen; AIs: Aromatase inhibitors.

## Competing interests

All authors declare that they have no competing interests.

## Authors’ contributions

YG and TC performed article search and drafted the manuscript. EL and WW participated in figures and table preparation. XW, JC and LT participated in the design of manuscript organization, manuscript refinement. JC and LT provided administrative support and funded experiments. All authors have contributed and approved the final manuscript.
